# RETRACTED: Wu et al. Silencing of *Ether à Go-Go 1* by shRNA Inhibits Osteosarcoma Growth and Cell Cycle Progression. *Int. J. Mol. Sci.* 2014, *15*, 5570–5581

**DOI:** 10.3390/ijms27010418

**Published:** 2025-12-31

**Authors:** Jin Wu, Daixing Zhong, Xijin Fu, Qingjun Liu, Liangqi Kang, Zhenqi Ding

**Affiliations:** 1 Department of Orthopaedics, The Affiliated Southeast Hospital of Xiamen University, Zhangzhou 363000, China; wuxinyu0102@163.com (J.W.); yuxin0506@163.com (X.F.); wujinzi1983@foxmail.com (Q.L.); 2 Department of Thoracic Surgery, The Affiliated Tangdu Hospital of Fourth Military Medical University, Xi’an 710038, China; xingxing.1230@hotmail.com

The Journal retracts the article “Silencing of ether à go-go 1 by shRNA inhibits osteosarcoma growth and cell cycle” [1] cited above.

Following the publication, concerns were brought to the attention of the Editorial Office regarding data presented in this article [1].

Adhering to our complaint procedure, an investigation was conducted by the Editorial Office and Editorial Board which confirmed an obvious error in the experimental design. While the authors collaborated within the investigation, this error could not be appropriately resolved. Consequently, the Editorial Board has lost confidence in the reliability of the findings and decided to retract this publication [1], as per MDPI’s retraction policy (https://www.mdpi.com/ethics#_bookmark30).

This retraction was approved by the Editor-in-Chief of the journal *IJMS*.

The authors agreed to this retraction. The author apologizes for the oversights in the preparation of the manuscript.
